# Discovery of a most remarkable cave-specialized trechine beetle from southern China (Coleoptera, Carabidae, Trechinae)

**DOI:** 10.3897/zookeys.725.21040

**Published:** 2017-12-29

**Authors:** Mingyi Tian, Sunbin Huang, Dianmei Wang

**Affiliations:** 1 Department of Entomology, College of Agriculture, South China Agricultural University, Wushan, Guangzhou, Guangdong, 510640, China

**Keywords:** aphaenopsian, Guangxi, ground beetle, troglobite

## Abstract

*Xuedytes bellus* Tian & Huang, **gen. et sp. n.** is described from a limestone cave in Du’an Karst of Guangxi, a kingdom of cavernicolous trechine beetles in southern China. From a morphological point of view, *Xuedytes* Tian & Huang, **gen. n.** seems to be the most extremely cave-adapted trechines in the world. Superficially, it looks much like *Giraffaphaenops* Deuve, 2002 in general body shape, in particular the structure of the prothorax, but simultaneously it is similar to *Dongodytes* (*s. str.*) Deuve, 1993, based on elytral characters, including chaetotaxy. Hence the new genus seems to represent a lineage intermediate between *Giraffaphaenops* and *Dongodytes* (*s. str.*).

## Introduction

The globe’s largest and highly varied karst landscapes that blanket nearly the entire area of southern China (Deharveng and Bedos 2012) are long known to host the richest diversity of cave-dwelling trechine beetles in the world. To date, 48 genera of cavernicolous trechines containing over 130 species have been recorded there (Deuve and Tian 2016, Tian et al. 2016, Zhao and Tian 2016, Fang et al. 2017, Huang et al. 2017). Many of them are morphologically highly cave-adapted, such as *Giraffaphaenops* Deuve, 2002, *Dongodytes* Deuve, 1993, *Uenotrechus* Deuve & Tian, 1999, *Pilosaphaenops* Deuve & Tian, 2008, *Sinaphaenops* Uéno & Wang, 1991 and *Shuangheaphaenops* Tian, 2017. As the cave beetle fauna of China is still poorly-known, it is hardly surprising that another highly peculiar species representing another new genus has been revealed in the country (Tian 2017).

In early August 2017, a cave biological survey carried out in Du’an Karst of northern Guangxi, southern China led to the discovery of a very peculiar species belonging to the subfamily Trechinae, family Carabidae. Moreover, it shows a number of most remarkable troglomorphic features amongst subterranean trechines generally. Superficially, its strongly elongated and slender body looks very much like that of a *Giraffaphaenops* species, especially due to the extremely elongated prothorax. However, *Giraffaphaenops* species are known from the Leye-Tianlin karsts of northwestern Guangxi, about 200 km away from Du’an Karst (Deuve 2002, Tian and Luo 2015). In contrast, the elytra in the new species are quite similar to those observed in *Dongodytes* (*s. str.*) Deuve, 1993, yet being much more strongly elongated. In addition, it has many other particular morphological characteristics, such as: (1) Head comparatively short, but sufficiently long and aphaenopsian, bearing multi-setiferous pores in frontal areas; (2) Right mandible edentate; (3) Prothorax extremely elongated; (4) Lateral margins of pronotum visible in fore part from above, while long erect setae on disc and two pairs of latero-marginal setae in middle portion present; and (5) Elytra extremely elongated, smooth and glabrous, with striae completely obliterated.

## Materials and methods

The beetles were collected in the cave using an aspirator, and kept in vials with 50% ethanol before study, except for a specimen put in a vial with 95% ethanol for molecular analysis.

Techniques and terminology are the same as in Tian (2017).

## Taxonomic treatment

### Xuedytes

Taxon classificationAnimaliaORDOFAMILIA

Tian & Huang gen. n.

http://zoobank.org/C8AACC92-F265-45CD-9F50-09DAB01ADFD0

#### Type species.

*Xuedytes bellus* Tian & Huang, sp. n.

#### Generic characteristics.

**Figure 1.**
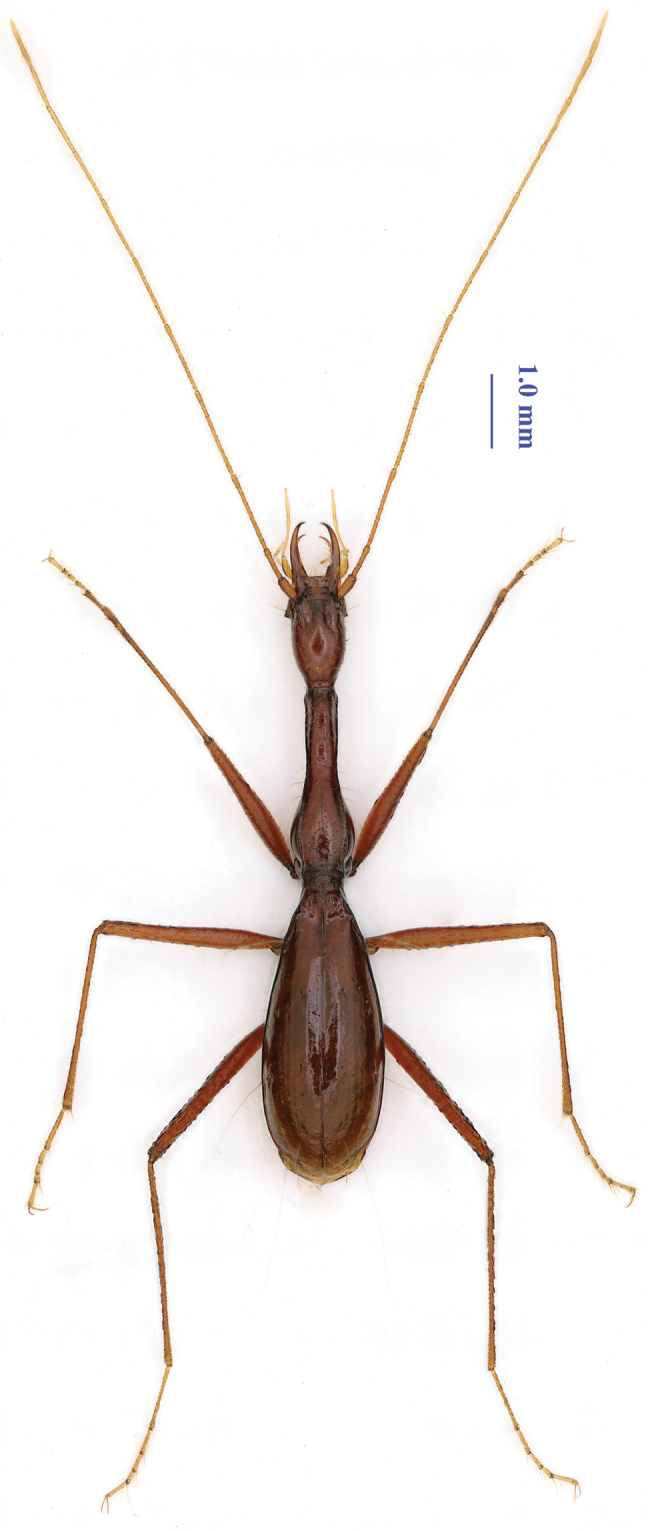
Habitus of *Xuedytes bellus* Tian & Huang, gen. et sp. n., holotype male.

**Figure 2.**
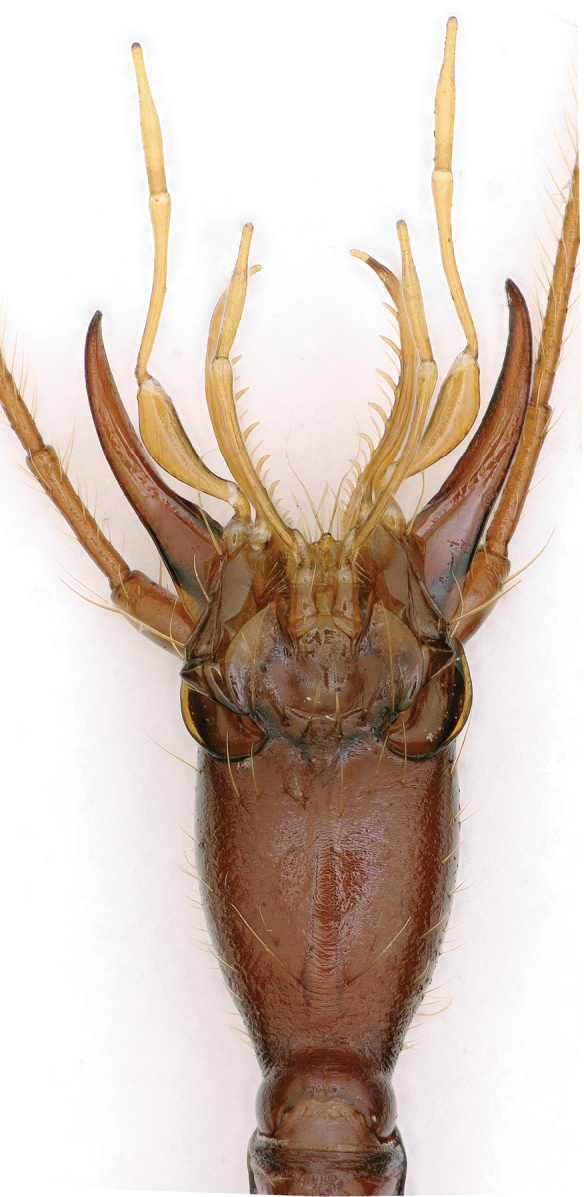
Head (ventral) of *Xuedytes bellus*, a paratype female.

Highly modified aphaenopsian trechines, body shape, in particular prothorax, similar to that in *Giraffaphaenops*, but elytra generally like in *Dongodytes* (*s. str.*) (Fig. 1); large-sized, with body (especially prothorax and elytra) and appendages thin and extremely elongated, eyeless and unpigmented; fore body part (head including mandibles, plus prothorax) much longer than, or as long as (excluding mandibles) elytra, respectively; body smooth; three pairs of frontal setiferous pores present on head; mandibles thin and elongated, feebly curved apically, longer than head width, right mandible edentate; labial suture completely missing; mentum bisetose on either side of tooth at base, base broadly concave; mental tooth simple, short and blunt at tip; submentum 8-setose; ligula bisetose at apex (Fig. 2); antennae very long, antennomeres 10 and 11 extending beyond elytral apices. Prothorax similar to that of *Giraffaphaenops*, wider than head, very strongly elongated, much longer than head including mandibles, propleura distinctly tumid in basal 1/3, visible from above; pronotum barrel-shaped, thin and distinctly elongated, lateral margins visible throughout from above, slightly narrower than head; hind latero-marginal setae absent, but two long latero-marginal setae plus two or three additional short setae present from middle to front. Elytra similar to those in *Dongodytes* (*s. str.*), narrow anteriorly and dilated posteriorly, side margins narrowly bordered throughout, shoulders lacking; striae virtually missing, only weakly traceable; two dorsal and the pre-apical setiferous pores present, each with a very long seta; chaetotaxy similar to that in *Dongodytes* (*s. str.*). Protibia smooth, without longitudinal sulcus; protarsomeres not modified in male. Ventrites VII bisetose apically in male, but quadrisetose in female. Male genitalia moderately sclerotized, small, strongly curved ventrally in lateral view, with a quite large and thin sagittal aileron; apical lobe wide and broad in dorsal view; parameres much shorter than median lobe, yet well-developed.

#### Discussion.

**Figure 3.**
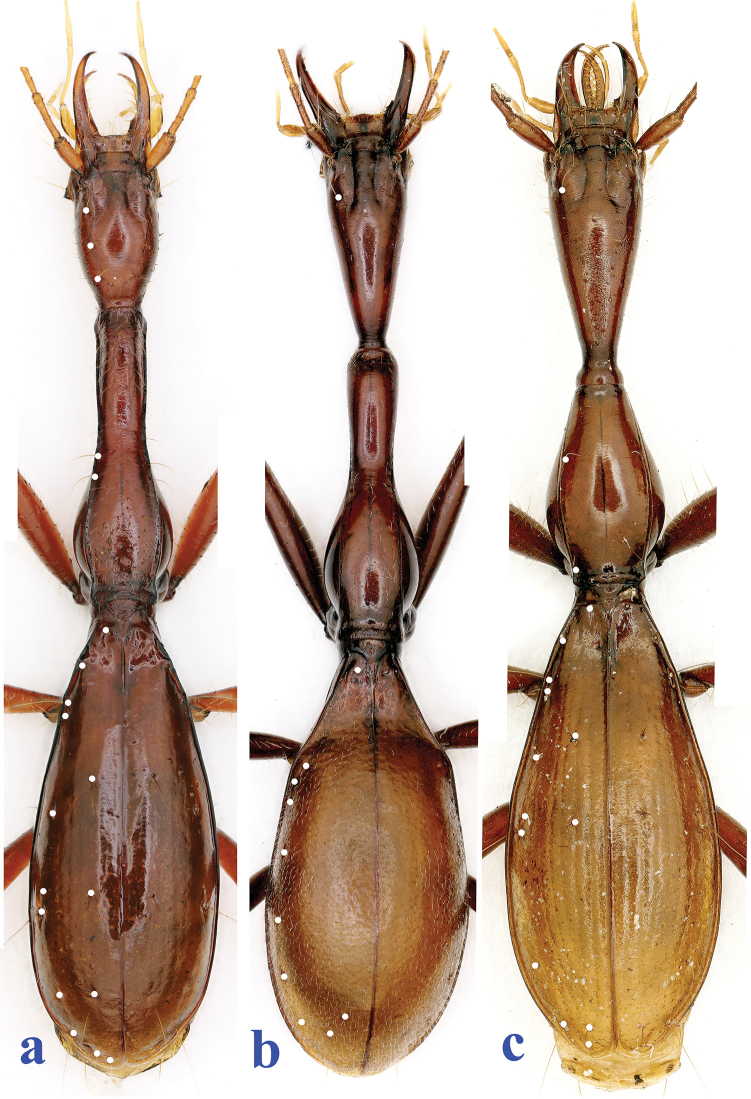
Habitus of three highly modified aphaenopsian beetles (chaetotaxy indicated by white points) **a** *Xuedytes bellus*, holotype male **b** *Giraffaphaenops clarkei* Deuve, 2002, male **c** *Dongodytes grandis* Uéno, 1998, male.

*Xuedytes* Tian & Huang, gen. n. is undoubtedly the most remarkable cavernicolous trechine genera as regards the extremely elongated prothorax and elytra. It may be considered as a lineage intermediate between *Giraffaphaenops* and *Dongodytes* (Fig. 3). Superficially it resembles *Giraffaphaenops* because of the similarly thin and strongly elongated body, especially the prothorax. Its elytra, however, are quite similar to those of *Dongodytes* (*s. str.*). The most striking character states of *Xuedytes* are as follows: (1) Prothorax much longer than head; (2) Elytra very narrow and strongly elongated; (3) Three pairs of frontal pores present on head; and (4) Right mandibular tooth obsolete.

Although *Xuedytes* is similar to *Giraffaphaenops*, there are several important differences: (1) Prothorax and elytra much more strongly elongated in *Xuedytes* than in *Giraffaphaenops*; (2) Head subquadrate, slightly convex laterally, not contracted posteriad in *Xuedytes* (vs. inversed triangular, with a well-marked neck constriction in *Giraffaphaenops*); (3) Entire lateral margins of pronotum visible from above in *Xuedytes* (vs. invisible from above in front half in *Giraffaphaenops*); and (4) Two pairs of latero-marginal setae present behind middle of pronotum in *Xuedytes* (vs. absent in *Giraffaphaenops*) (Fig. 3).

Apart from the differences in prothoracic features, *Xuedytes* is easily distinguished from *Dongodytes* (*s. str.*) by the following characteristics: (1) Head thicker and broader, not narrowed posteriad in *Xuedytes* (vs. thinner and evidently narrowed posteriad, forming a long and distinct neck constriction in *Dongodytes*); (2) Three pairs of frontal pores present in *Xuedytes*, instead of only one pair in *Dongodytes*; (3) Elytral striae completely obliterated in *Xuedytes* (vs. partially visible in *Dongodytes*) (Fig. 3).

**Figure 4.**
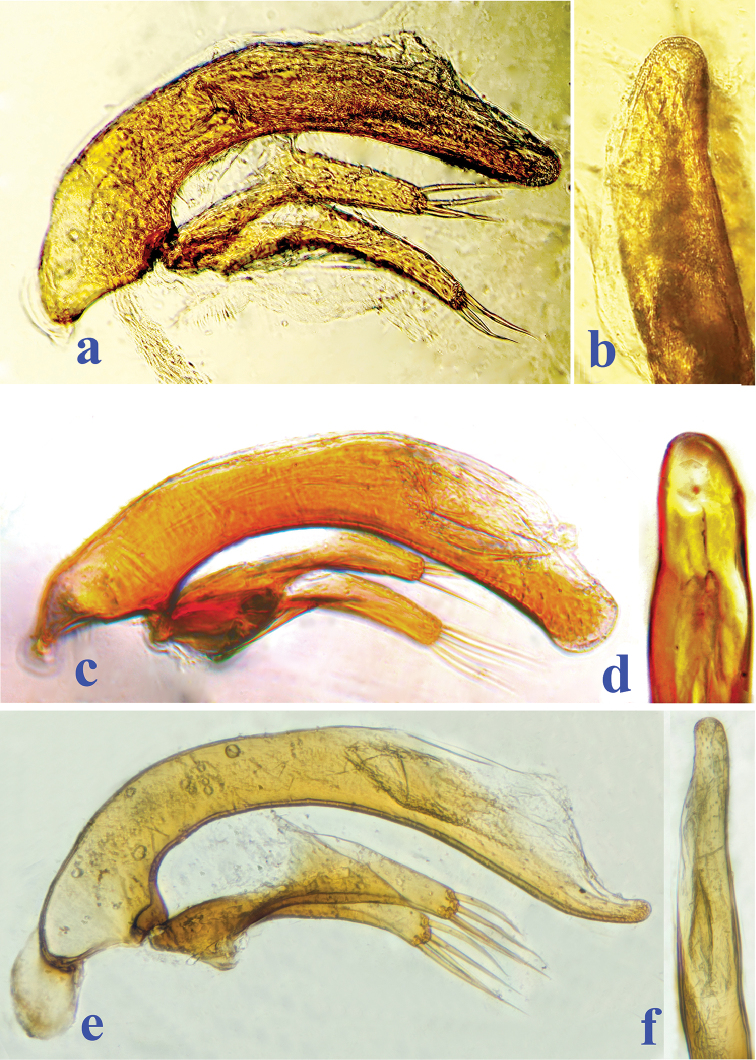
Male genitalia of three highly modified aphaenopsian beetles, median lobe and parameres in lateral, and apical lobe in dorsal views, respectively **a, b** *Xuedytes bellus* **c, d** *Giraffaphaenops yangi* Tian & Luo, 2015 **e** *Dongodytes grandis* Uéno, 1998.

Furthermore, differences between the new genus and both *Giraffaphaenops* and *Dongodytes* are also evident regarding the structure of the male genitalia (Fig. 4). The median lobe of the aedeagus is shorter in *Xuedytes*, but thicker, especially so at the base, with a thinner, almost transparent sagittal aileron.

#### Etymology.

“Xue + dytes”. “Xue” in Chinese means “cave”, to indicate that the beetles are cavernicolous. Gender masculine.

#### Generic range.

China (Guangxi Zhuang Autonomous Region).

### Xuedytes bellus

Taxon classificationAnimaliaORDOFAMILIA

Tian & Huang sp. n.

http://zoobank.org/13622878-92B3-43B7-B253-426CE076E37F

**Figure 5.**
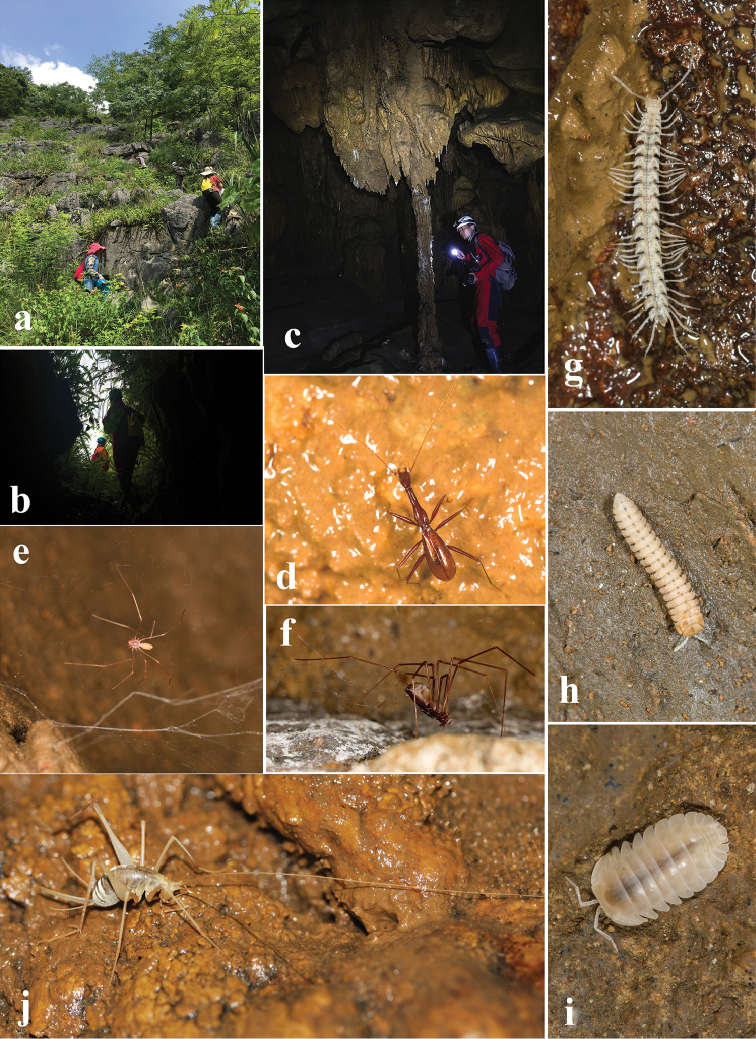
Cave II, southeastern Du’an, the type locality of *Xuedytes bellus*, and sympatric cave animals **a, b** cave environs and opening **c** a chamber in the cave where the beetles were collected **d** a running beetle in cave **e, f** cave spiders **g, h** cave millipedes **i** a cave woodlouse **j** a cave cricket.

Figs 1 , 2 , 3 , 4 , 5

#### Holotype.

Male, Cave II, southeastern Du’an Yao Autonomous County, Hechi Shi (=Prefecture), northern Guangxi Zhuang Autonomous Region, southern China, VIII-08-2017, leg. Mingyi Tian, Sunbin Huang, Dianmei Wang and Mengzhen Chen; paratypes: 2 males and 1 female, IBID. All type material is deposited in the insect collection of South China Agricultural University, Guangzhou.

#### Diagnosis.

A large-sized, blind, cave-adapted trechine, remarkably modified morphologically, with both prothorax and elytra highly elongated and slender so that body five times longer than wide, antennae slightly shorter than body including mandibles, extending beyond elytral apices; head, pronotum and base of elytra covered with sparse erect setae. Habitus as in Figs 1 and 3.

#### Description.

Length: 8.3–9.0 mm (from apex of right mandible to elytral apex) or 7.5–8.6 mm (from labrum to elytral apex); width: 1.4–1.5 mm.

Body yellowish brown, but antennae, palps and tarsi pale; strongly shining; head, pronotum and base of elytra covered with rather long and sparse setae, other parts of elytra glabrous, underside of fore body excluding pleurae, meso- and metasterna pubescent, abdominal ventrites densely pubescent; microsculptural engraved meshes transverse striate on head, pronotum and elytra; fore body very strongly elongated, much longer than elytra, (HLm+PrL)/EL = 1.55–1.60.

Head oblong subquadrate, much longer than wide, HLm/HW = 3.02–3.03, HLl/HW = 2.02–2.04; genae fairly well developed, broadly dilated on sides, widest at about middle of head from neck to clypeal margin, gradually tapered posteriorly; frons and vertex moderately convex, frontal furrows moderately defined, strongly diverging posteriorly, ending level with middle frontal pores; clypeus transverse, 6-setose; labrum transverse, frontal margin nearly truncate, 6-setose; three pairs of frontal setiferous pores present; mentum and submentum completely fused, mentum bisetose on either side of tooth at base, mental tooth short and blunt at apex, basal fovea broadly concave; submentum 8-setose; palps thin and very slender, glabrous except for labial palpomere 2 which is bisetose on inner margin; 2^nd^ labial palpomere 1.40 times longer than 3^rd^; 3^rd^ maxillary palpomere 1.25 times longer than 4^th^; suborbital pore much closer to base than to submentum (Fig. 2). Antennae thin and very long, 1^st^ antennomere shortest and stoutest, 4^th^ longest, 11^th^ longer than 10^th^, length ratios of antennomere 1 to 11 as 1.00 / 1.36 / 3.07 / 3.55 / 2.77 / 2.81 / 2.52 / 2.10 / 1.94 / 1.65 / 1.94.

Prothorax (Fig. 3a) much longer than head including mandibles, PrL/HLm = 1.15–1.17, PrL/HLl = 1.70–1.72, widest at about 1/5 off base, nearly 3 times as long as wide, PrL/PrW = 2.94–2.95, slightly wider than head, PrW/HW = 1.18–1.19, evidently wider than pronotum, PrW/PnW = 1.27–1.30. Pronotum very strongly elongated, tube-like in front half which is narrow and nearly parallel-sided; convex at about basal 1/5 where the widest point lies, then gently sinuate before hind angles which are obtuse and rectangular, fore angles rounded; nearly four times longer than wide, PnL/PnW = 3.84, slightly narrower than head, PnW/HW = 0.91–0.92, base slightly concave, wider than front, PbW/PfW = 1.27, front convex; lateral sides finely bordered throughout, base and front unbordered; basal latero-marginal setae absent, two latero-marginal setae present in about middle portion, with three or four additional short setae in fore part; disc strongly convex in front and moderately convex in basal half, deeply concave a little before middle; median line clear, but shallow, basal transverse impression well-marked, short; scutellum fairly large.

Elytra (Fig. 3a) very strongly elongated ovate, much longer than wide, EL/EW = 2.46; longer than prothorax, EL/PrL = 1.58–1.59, almost twice as wide as prothorax, EW/PrW = 1.90–1.91; distinctly dilated posteriorly, widest at about 3/5 of elytra off base, lateral sides smooth, not ciliate, finely bordered throughout, marginal gutters well-marked; disc distinctly convex, but evidently concave near base; striae virtually missing, yet more or less traceable, intervals moderately convex. Chaetotaxy (Fig. 3a): dorsal pores with stout and long setae, two dorsal setiferous pores present on 3^rd^ stria at about 1/3 and 3/5 of elytra off base, respectively; pre-apical pore at about apical 1/6 of elytra, much closer to elytral suture than to apical margin; basal pore located between scutellum and marginal gutter; marginal umbilicate pores not aggregated, pores 1–3 and 10 near marginal gutter, other pores distant from gutter; humeral groups with pores 1 and 4 widely isolated, 2 and 3 close to each other, distance from pore 4 to 3 subequal to 5; middle group closely spaced, distance of pore 5 and 4 subequal to that of pore 5 and 6; apical group composed of four pores.

Legs slender and long, bearing short pubescence; fore and middle femora sparsely setose; fore tibia smooth, with neither a longitudinal furrow nor a sulcus; 1^st^ tarsomere shorter than, slightly longer than, and much longer than 2^nd^–4^th^ tarsomeres combined in fore, middle, and hind legs, respectively.

Male genitalia (Fig. 4a, b). Aedeagus quite small and short, distinctly curved ventrally in middle portion in lateral view, then broad at apex; inner sac with a fairly large copulatory piece, the latter about 1/3 as long as median lobe; base quite large, open ventrally, with a hyaline sagittal aileron; in dorsal view, apical lobe fairly stout, slightly sinuate on right side, rounded at apex. Parameres short and quite elongated, each bearing four long apical setae.

#### Etymology.

“*Bellus*”, in Latin meaning “beautiful”, to refer to this beautiful aphaenopsian beetle.

#### Distribution.

China (Guangxi: Du’an). Known only from Cave II.

This cave maintains a natural condition, opening on a small hill on the northern bank of the Hongshui River. The entrance is surrounded by dense bushes and not readily accessible (Fig. 5a, b). The total length of the cave is still unknown, but said to be about 200 m, according to local people. It is sufficiently wet inside the gallery and is good for cave fauna. The beetles were found running on walls and stalactites (Fig. 5c, d), sympatric with spiders (Fig. 5e, f), millipedes (Fig. 5g, h), woodlice (Fig. 5i) and crickets (Fig. 5j).

## Supplementary Material

XML Treatment for Xuedytes

XML Treatment for Xuedytes bellus
